# Large Language Models and OpenLogos: An Educational Case Scenario

**DOI:** 10.12688/openreseurope.17605.1

**Published:** 2024-06-05

**Authors:** Andrijana Pavlova, Branislav Gerazov, Anabela Barreiro

**Affiliations:** 1 “Krste Misirkov”, UKIM, Institute of Macedonian Language, Skopje, North Macedonia; 2 UKIM, Faculty of Electrical Engineering and Information Technologies, Skopje, North Macedonia; 3 Human Language Technologies Laboratory (HLT), Instituto de Engenharia de Sistemas e Computadores Investigacao e Desenvolvimento em Lisboa, Lisbon, Lisbon, Portugal

**Keywords:** Natural Language Generation, Large Language Models, Generative Artificial Intelligence, Multi3Generation COST Action, OpenLogos, Education.

## Abstract

Large Language Models (LLMs) offer advanced text generation capabilities, sometimes surpassing human abilities. However, their use without proper expertise poses significant challenges, particularly in educational contexts. This article explores different facets of natural language generation (NLG) within the educational realm, assessing its advantages and disadvantages, particularly concerning LLMs. It addresses concerns regarding the opacity of LLMs and the potential bias in their generated content, advocating for transparent solutions. Therefore, it examines the feasibility of integrating OpenLogos expert-crafted resources into language generation tools used for paraphrasing and translation. In the context of the Multi3Generation COST Action (CA18231), we have been emphasizing the significance of incorporating OpenLogos into language generation processes, and the need for clear guidelines and ethical standards in generative models involving multilingual, multimodal, and multitasking capabilities. The Multi3Generation initiative strives to progress NLG research for societal welfare, including its educational applications. It promotes inclusive models inspired by the Logos Model, prioritizing transparency, human control, preservation of language principles and meaning, and acknowledgment of the expertise of resource creators. We envision a scenario where OpenLogos can contribute significantly to inclusive AI-supported education. Ethical considerations and limitations related to AI implementation in education are explored, highlighting the importance of maintaining a balanced approach consistent with traditional educational principles. Ultimately, the article advocates for educators to adopt innovative tools and methodologies to foster dynamic learning environments that facilitate linguistic development and growth.

## Plain language summary

Large Language Models boast advanced text generation quality and capabilities, often surpassing those of humans. However, they also pose significant challenges when used without proper expertise or care. In an educational context, the examination of language generation tools and their use by students is vital for establishing guidelines and a shared understanding of their ethical usage. This article explores several aspects of language generation within an educational context, and showcases the potential use of OpenLogos resources, provided within the framework of the Multi3Generation COST Action (CA18231) in language study and their integration into language learning tools, such as paraphrasing (monolingual) and translation (bilingual or multilingual). This article emphasizes the importance of leveraging OpenLogos in education, especially in language learning or language enhancement contexts. By embracing innovative tools and methodologies, educators can nurture a dynamic and enriching learning environment conducive to linguistic growth and development.

## Introduction

Language plays an omnipresent role in every aspect of human existence and development, gaining heightened importance, especially in the context of education. The technological impact of Natural Language Generation (NLG) tools in education raises several challenging questions, such as: (i) Can these tools aid students in improving their overall literacy and education? (ii) Could these tools potentially result in a decrease in students’ critical thinking abilities and their capacity to express themselves and participate in societal discourse? (iii) Is society and the educational system prepared to accept the integration of NLG tools into the educational setting, especially within the classroom?

While Large Language Models (LLMs) have advanced text generation, they also pose scientific challenges. This article provides an overview of the current state of NLG and examines the benefits and drawbacks of using LLMs, particularly in educational contexts. Due to the opaque nature of LLMs ("black box"), understanding their inner workings can be difficult, often resulting in heavy reliance on the system’s outputs for learning purposes, especially on its biases. On the other hand, resources like OpenLogos offer the potential to create "glass box" systems, providing transparency into the generative process. These resources were made available online through GitHub ^1^ as part of the Multi3Generation COST Action (CA18231) initiative ^1^. Multi3Generation is dedicated to collaborative efforts aimed at advancing NLG research for societal benefit (and not harm), including its application in educational aid, to support learning and language acquisition. The initiative also promotes the development of alternative or supplementary models that draw inspiration from the Logos Model, emphasizing inclusivity by considering resources and methods from various linguistic models. These innovative human-centred models should prioritize improving the transparency, adaptability, and traceability of Natural Language Processing (NLP), particularly in NLG systems used for applications such as Machine Translation (MT), paraphrasing, and, notably, Language Learning.

The remainder of this article is structured as follows: Section 2 provides an overview of the role of NLG within an educational context. It highlights the current challenges and drawbacks associated with the usage of LLMs and AI-generated content among students. The section emphasizes the need for well-defined and structured guidelines for the appropriate integration of LLMs in educational settings. Section 3 offers an overview of the various roles that LLMs and AI can play in education and proposes a model for integrating LLMs and AI-generated content. Section 4 discusses the potential role of OpenLogos in AI-assisted education and its significance in inspiring the development of ethical systems that prioritize proper documentation and protect the rights of their creators and developers. Section 5 presents the potential benefits that GenAI, LLMs and OpenLogos bring to Education. Section 6 delves into the limitations and ethical considerations surrounding the implementation of AI in education. Finally, Section 7 presents the conclusions drawn from the discussion and outlines potential avenues for future research.

## Natural Language Generation in an educational context

The capability of generating language is the most important manifestation of superior intelligence that distinguishes humans from other animals. For instance, a parrot can mimic words without understanding what they mean. One of the basic skills that children learn in language acquisition is to extend their vocabulary with new words and understand the contexts in which these words are used. The difference between the parrot and the child in producing words is called the "faculty of language" ^2^ ^2^, which is non-existent in non-human animals.

The recent advances in AI and NLG have led to the development of advanced LLMs. These models use deep learning techniques and massively large data sets to communicate in a human-like manner. They can computer-‘understand’, summarize, generate, and predict different types of content (text, images, videos, graphical representations, code, etc.). LLMs, especially the advanced state-of-the-art chatbots, such as ChatGPT, show remarkable proficiency in many languages, including Macedonian, and Portuguese.

The main purpose of the LLMs is to serve us as assistants. LLMs are multilingual. They can generate short or long responses on various topics from different domains. LLMs can summarize given texts, organize, and classify data, translate texts in different languages ^3, 4^. Upon our request, they can receive information in one language and respond into another language ^5^. LLMs can write explanations, slogans, songs, stories, and drama, they can imitate styles, write code, devise games, generate questions for exams and surveys, create tabular and graphical representations, solve mathematical or logical problems. Advanced state-of-the-art LLMs, like ChatGPT, can generate videos, images, graphics, and code. They can read and extract text from images or .pdf files and transcribe voice messages. Additionally, LLMs can recognize and respond to the emotional tone in a text. Due to the patterns learned from the training data, they can generate responses that would be typical for a human in a given emotional context ^6^. LLMs also have weaknesses and limitations – they are prone to hallucinations ^7– 9^, sometimes they can generate biased, inappropriate, or inaccurate content and they are not so proficient in low or mid-resource languages ^6^. For low or mid-resource languages, LLMs, like foreign language users, often employ compensatory strategies ^6^, such as:

- Analytical strategies (circumlocution, description, paraphrasing)
- Holistic strategies (superordinate, subordinate, or co-ordinate concept)
- Morphological creativity (creativity in word formation, coining non-existing words)
- Strategy of transfer (borrowing, literal translation, foreignizing, overgeneralization, switching or mixing language codes), etc. ^5, 10, 11^.

Besides the limitations, LLMs have enormous computational power. They are trained on massive data sets in multiple languages and knowledge domains. LLMs effectiveness depends on how well users can communicate their needs. Prompting well means knowing how and what to ask. It is important to be explicit and to provide clear explanations or information within the token limit. While prompting and guidance are undoubtedly important aspects of education, the essence of education is the transfer of knowledge, fostering critical thinking skills, encouraging curiosity, and nurturing personal development. A responsible use of AI in an educational environment can complement the broader educational process. It is essential to maintain a balanced perspective and recognize that while AI can enhance and supplement educational experiences, it cannot replace the fundamental principles and goals of education.

The use of LLMs in education presents a spectrum of potential outcomes, encompassing both advantageous and detrimental effects. Table 1 provides a concise overview of the positive and negative impacts associated with their incorporation in educational settings, which will be explained in detail in subsection 2.1– subsection 2.2, contrasted in subsection 2.3, and summed up in subsection 2.4.

**Table 1.** Negative and positive effects of LLMs in education.

Positive Effects	Negative Effects
Large learning resources	Bias and fairness concerns
Support grading and providing feedback	Lack of personalization
Language proficiency improvement	Overemphasis on memorization
Adaptive learning platforms	Privacy concerns
Innovative teaching methods	Misinformation and hallucinations
–	Laziness, lack of creativity and reduction of critical thinking
–	Cheating and plagiarism

### Positive effects of LLMs in education

LLMs can offer numerous positive impacts on education. Firstly, they facilitate the development of extensive learning resources, including multilingual and multimodal materials, to enrich the learning process. However, there’s often a gap between intention and achievement due to factors like inadequate training or knowledge. Secondly, LLMs streamline grading and feedback processes, benefiting both students and instructors by providing timely and constructive feedback. Thirdly, they contribute to improving language proficiency through technology-driven platforms, although achieving proficiency in speaking, listening, and reading may require additional factors. Additionally, LLMs serve as the foundation for adaptive learning methods, leveraging technology to personalize instruction and enhance learning outcomes. Lastly, they enable innovative teaching approaches that integrate language and machine learning technologies to optimize the teaching process. However, realizing these benefits depends on further research and integration into educational practices. Making this possible and attaining satisfactory outcomes depends on numerous factors that are currently under investigation, primarily due to the limited integration into mainstream educational practices and the need for further research to solidify results using cutting-edge techniques.

### Negative effects of LLMs in education

When considering the negative impacts of LLMs in education, several key aspects need attention. Firstly, there’s the issue of bias and fairness concerns, stemming from biases in training data and algorithms, leading to discriminatory outcomes and perpetuation of stereotypes. Secondly, the lack of personalization in LLMs hampers tailored interactions and may result in less engaging experiences, especially when users seek varying levels of formality or technicality. Thirdly, an excessive focus on memorization sidelines deeper understanding and critical thinking, hindering meaningful learning outcomes. Privacy concerns also arise, encompassing worries about data security, consent, and legal compliance in handling personal information ^3^. Moreover, misinformation and hallucinations ^4^ pose risks of disseminating false or nonsensical content, challenging the credibility of LLMs and educational integrity. Additionally, LLMs can foster laziness, reduce creativity, and diminish critical thinking skills, as users may over-rely on the models for generating content without engaging in independent thought or analysis. Finally, cheating and plagiarism facilitated by LLMs undermine academic integrity, needing proactive measures to promote ethical behavior and uphold academic standards. Addressing these challenges requires a multifaceted approach involving education, policy, technology, and ethical considerations.

### Striking a balance between the positive and the negative impacts of LLMs in education

When evaluating the impact of LLMs in education, it is crucial to weigh both their advantages and disadvantages. Positively, LLMs contribute to enhanced learning resources and streamline grading and feedback processes, facilitating efficient assessment and improvement for students. They also hold promise in improving language proficiency through personalized learning experiences and adaptive platforms. However, there are notable concerns regarding the lack of transparency in LLM operations, hindering users’ understanding of their functionality and potentially fostering dependency without true comprehension. Moreover, while LLMs excel in written language skills, they may fall short in addressing other language aspects like speaking and listening, which require nuanced practice. In conclusion, while LLMs offer substantial benefits in education, including resource enhancement and personalized learning, their implementation should be approached cautiously, ensuring transparency and comprehensive learning strategies.

### Summing up

Up to this date, LLMs and other generative models are not entirely adequate for language acquisition as they do not process or reproduce language as humans do, with the diversity and idiosyncrasy that is characteristic of each language, language varieties, dialects, etc. The use of NLG models by students and, eventually, in the classroom environment should be handled with care. Currently, without proper guidance or training, ChatGPT may promote laziness, lack of creativity, and reduction of critical thinking in inexperienced and untrained students. It can also be employed for engaging in plagiarism-related activities. Additionally, it can feed students with critical knowledge errors (in case of uncontrolled outputs) that they are unable to detect. As an instrument used in the field of education by the young generation, when used without supervision, tools such as ChatGPT may easily become “cheating tools” instead of tools to foster intellectual development and qualitative reasoning ^12– 14^. Many newspapers’ articles report the use of ChatGPT to cheat on school assignments ^5, 6, 7, 8, 9, 10, 11^. The potential integration of LLMs in education, in general, or for specific purposes (second language acquisition, Vocational Education and Training (VET), translation, assistive technology for students with learning disabilities, among others) has been a point of exploration and debate since 2021. On the positive side, many studies have also shown that the deployment of these generative models in educational settings, with proper pedagogical guidance, can be valuable ^6, 15– 19^. If used properly, LLMs can be beneficial for empowering students’ writing skills, fostering innovation, and nurturing independent and even critical thinking. These models aid as translating tools and can help students and teachers effectively navigate language barriers ^20^. Besides this, they can be used as alternative communication systems for students with learning disabilities or students with neurodevelopmental disorders such as ADHD, ADD, and Autism ^21– 23^.

LLMs have an enormous capacity for revolutionizing education, but there are significant challenges such as hallucinations, biases, reasoning errors, and weaker performances in low or mid-resource languages (such as Macedonian) among others, which represent a critical obstacle for their application in education ^6, 7, 9, 24^. Hallucinations in LLMs refer to the generation of content that is inaccurate, irrelevant, or inconsistent with the input data. Such issues are often arising from the training data’s quality and the models’ interpretative limits. Besides this, there are ethical and legal concerns associated with the use of LLMs in education, which need to be addressed. However, by proactively tackling these issues, we could pave the way for their application in education, guaranteeing educational experiences that are inclusive, ethical, and impactful. To sum up, the deployment of LLMs and chatbots in education offers significant advantages and can be generously beneficial. Training teachers, educators, and students on how to appropriately use LLMs and other generative models is essential for unlocking the transformative potential of LLMs. With a deeper understanding of how LLMs work, and their fundamental limitations, teachers and students can make more informed decisions about how LLMs could be used in an educational context without further exacerbating the existing social disparities.

## LLMs integration in education: a case scenario

**Figure 1.**
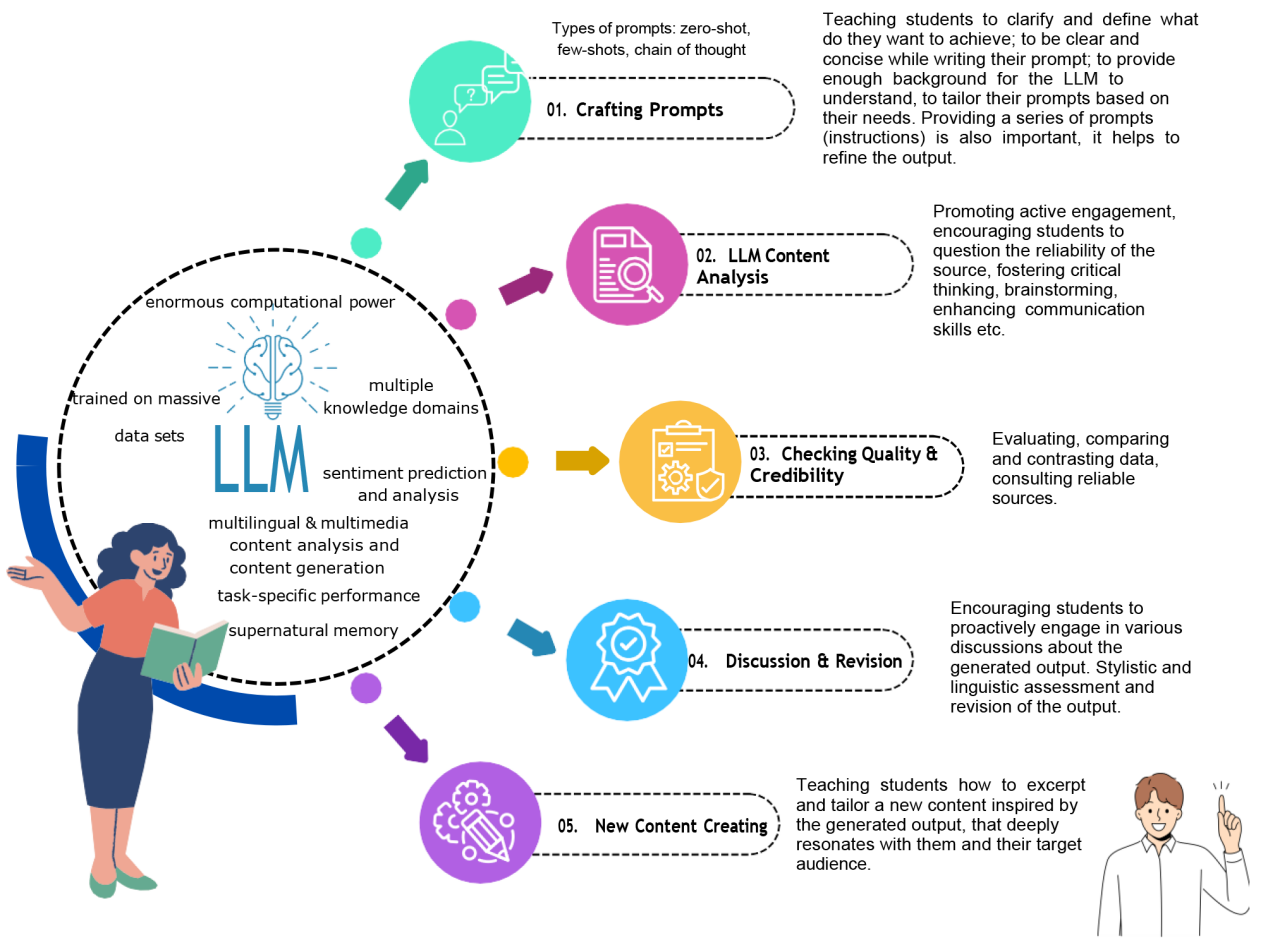
Model of LLMs/GenAI integration in education: structured interaction scenario.

The implementation of generative AI technologies (GenAI tools) in education represents a major turning point, demanding a re-evaluation and enhancement of the traditional skill set of teachers ^25^. As for the implementation of GenAI in education and the appropriate age limit, UNESCO highlights that the users should be at least 13 years old, but there are still debates about whether the age limit should be raised to 16 years old ^16, 22^ ^12^. Soon, we expect hybrid educational methods to be implemented (traditional teaching or human-centred methods) with AI-assisted teaching. First and foremost, teachers and educators must master the fundamental concepts of generative tools and be equipped with practical skills to integrate them effectively in the classroom. The role of the teacher will be to stimulate the students to use the LLMs and other generative models properly that will foster their curiosity and critical thinking. In Figure 1 we illustrate a basic model of LLMs integration in education ^13^.

The proposed model was inspired by the study of ^22^. It represents a structured educational scenario, with three participants: teacher, LLM (AI), and student(s). The main role of the teacher is to be a moderator in this process, while the students should take an active role in each stage of the process. Prior to the use of the LLM model, the teacher or some AI educator should introduce the students to the basics of LLMs, emphasizing their strengths and weaknesses. Then, step by step, they should present the types of prompts (zero prompt, few-shot prompt, and chain of thoughts), giving an example for each type of prompt.

1. The first step is to train students to master the art of questioning and prompting ^14^. The teacher should present a specific problem or topic related to a particular subject and ignite curiosity within the students. Then, they should encourage them to use the AI chatbot to delve deeper into the subject, introducing them to the basic steps of prompting. Students should:
  • Provide enough background for the LLM to understand the request.
  • Specify their personal preferences (ask the LLM to act as [role-play], specify your point of interest, specify the profile of the audience, specify the style, etc.).
  • Specify the format of the output (long/short answer, unspecified, answer by example, provide tabular or graphical representation, create an image, write a summary, extract text from an image, create a story inspired by an image, transcribe a voice message, make questions, create a survey on a specific topic, etc.).
  • Provide a series of prompts (ask the LLM to generate additional solutions, or to paraphrase the previous output, etc.).
  Students should explore different ways of crafting prompts (queries). Providing a series of prompts (instructions) is also important as it can help refine the output. This activity will empower students to take an active role, explore, and become more curious and open-minded. Prompting and questioning provoke active engagement of the students in the subject matter.
2. In the second step, the role of the teacher will be to encourage the students to share their opinions, ideas, or doubts about the generated output. Students should proactively engage in discussions by analyzing, and evaluating the content of the output. This type of activity will foster brainstorming, critical thinking, collaborative decision-making, and active engagement in the learning process. Moreover, it will promote the process of learning from AI’s mistakes.
3. The third step is the evaluation of the generated output. Students should check the quality and credibility of the output. The role of the teacher will be to provide them with information about reliable sources on the subject matter (consulting books, searching, and consulting papers from Google Scholar, etc.). He/she must warn the students that LLMs are not reliable sources, so they must check the information to avoid negative (knowledge or language) transfer from the output. This activity will promote the benefits of the process of evaluation, research, and critical thinking. Students should master the art of critical reading and recognizing misinformation/fake (false) information.
4. The fourth step is stylistic and linguistic assessment and revision of the output. The role of the teacher is to encourage the students to proactively engage in various discussions about the structure and the language of the generated output.
5. The final (fifth) step is writing an essay or paper on the subject matter using the knowledge gained through the previous steps. The role of the teacher will be to teach them how to tailor new content inspired by the AI’s output that will resonate with them and their target audience. Students should master the art of text editing.

Some universities have offered guidelines for citing LLMs ^17^ ^15^, while other institutions, like the publisher Cambridge University Press firmly state that the AI does not meet the requirements for authorship ^19^ and offer guidelines that “will help researchers to use generative artificial intelligence (AI) tools like ChatGPT while upholding academic standards around transparency, plagiarism, accuracy and originality” ^18^ ^16^. The principles of the Cambridge University Press guidelines are as follows ^19^ ^17^:

- The use of AI to generate content for submissions must be declared and clearly explained in the text when submitted to Cambridge Open Engage.
- AI does not meet the Cambridge requirements for authorship, given the need for accountability. AI and LLM tools may not be listed as authors on any content on Cambridge Open Engage.
- Authors are accountable for the accuracy, integrity, and originality of their research work, including for any use of AI.
- Any use of AI must not breach the Cambridge Open Engage Terms of Submission. Scholarly works must be the author’s own and not present others’ ideas, data, words, or other material without adequate citation and transparent referencing.

We agree with the accountability of authors for the accuracy, integrity, and originality of their research work, even when utilizing AI. The model presented here is versatile and can be implemented by teachers at various educational levels, including high school, college, and university, with the flexibility to tailor it to different subject matters.

## Using OpenLogos for educational purposes

The Human Language Technologies Laboratory (HLT) at INESC-ID has been instrumental in driving forward NLG tools and resources, particularly through its involvement in initiatives like the Multi3Generation project, focusing on paraphrasing ^26^ and translation ^27^, using OpenLogos resources provided by the COST Action. One outcome of these efforts is the development of the eSPERTo paraphrasing tool ^28^, which enables users to generate paraphrases, or alternative expressions, of text. These paraphrases are stored in "paraphrasaries" ^29, 30^, which are sophisticated extensions of dictionaries suitable for both monolingual and multilingual applications.

OpenLogos ^31, 32^, initially developed as a machine translation system by Logos Corporation ^18^, has transitioned into an educational tool within the field of NLP. With its emphasis on semantic understanding and linguistic analysis, OpenLogos presents a distinctive approach to language learning and comprehension. In the realm of machine translation, the integration of high-quality paraphrasing knowledge is crucial for achieving precise translations. However, there remains potential for refinement in alignment tools and methodologies to establish degrees of equivalence and broaden paraphrastic units. These advancements could enhance the accuracy of both paraphrasing and translation, aligning with the societal objectives envisioned for NLG technologies. Below are several potential avenues for the evolution of OpenLogos as an educational tool:

1. **Language Learning**: OpenLogos can provide students with the opportunity to engage with real-world language data and analyze linguistic structures. By exploring translations and paraphrases, students can deepen their understanding of grammar, syntax, and vocabulary in different languages.
2. **Translation Studies**: OpenLogos can offer a rich source of translated texts, making it an ideal resource for students studying translation. Students can compare translations produced by OpenLogos with human translations to identify patterns, challenges, and strategies in the translation process.
3. **Computational Linguistics**: OpenLogos can expose students to the inner workings, i.e., the underlying algorithms and techniques used in NLP, and can gain insights into machine learning, artificial intelligence, and computational linguistics.
4. **Linguistic Research**: Researchers and linguists can leverage OpenLogos to study language variation, language change, and linguistic typology. The vast amount of language data available in OpenLogos enables researchers to conduct empirical studies and analyze language phenomena across different languages and language families.
5. **Language Teaching and Assessment**: OpenLogos can be integrated into language teaching curricula to supplement traditional teaching methods. Educators can use OpenLogos to create language learning activities, exercises, and assessments tailored to the needs of their students.

**Figure 2.**
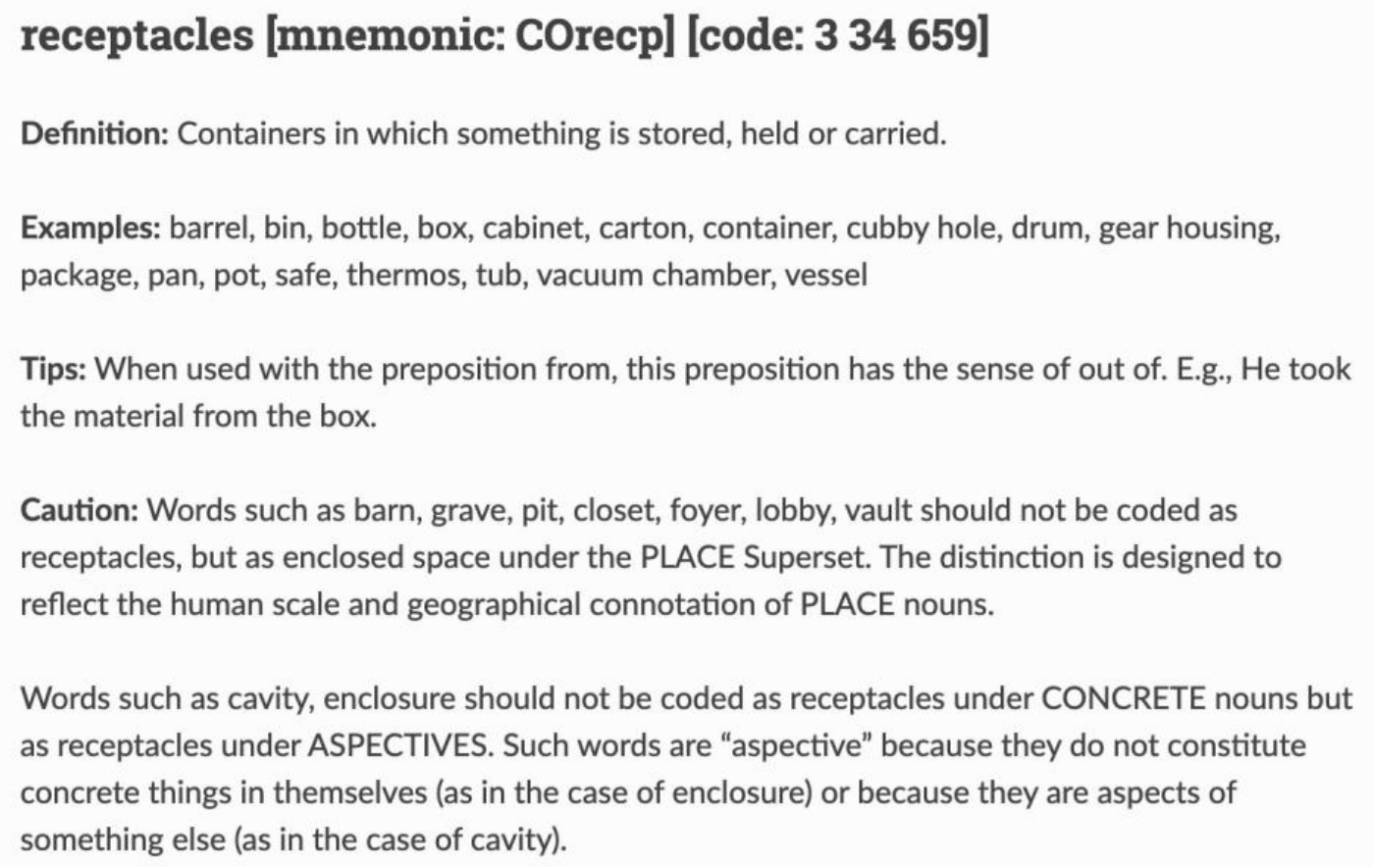
OpenLogos coding of concrete nouns (CO): receptacles (recp).

OpenLogos relies on the Semantico-Syntactic Abstraction Language (SAL) – a representation language that enables the computer to process sentences both at the level of word meaning (semantics) and sentence structure (syntax) ^19^. In Figure 2 we present an example table for OpenLogos coding of a concrete noun of the type ‘receptacles’ ^20^. Unlike word embeddings used in Deep Learning models, which are derived from extensive datasets, the OpenLogos coding is meticulously crafted by human linguistic experts, and it undergoes continuous refinement through a trial-and-error process based on its success or failure in generating desired outputs. The table includes the definition of the word (the subset), lists examples that belong to it, and shares tips and gotchas.

Indeed, if developed through collaborative efforts, OpenLogos has the potential to become a versatile and impactful educational tool. It can facilitate language learning, encourage linguistic exploration, and enhance computational reasoning skills. By offering access to genuine language data and sophisticated language processing features, OpenLogos can elevate the educational experience and equip students and researchers with valuable resources for their language related pursuits. The OpenLogos resources offer a valuable tool for paraphrasing and translating texts from English and German into French, Italian, Spanish, and Portuguese. Several articles have been written in previous years illustrating the Model ^33^, sharing linguistic resources ^34^, and comparing translations by OpenLogos to those generated by Google Translate ^35^, among other experiments published in random articles. Similarly, several articles have described the work done on paraphrasing over the years using initial OpenLogos resources ^26, 28, 36, 37^, among others, including recent experiments comparing paraphrases generated by the eSPERTo paraphrasing tool for Portuguese to paraphrases generated by ChatGPT ^38^. By using OpenLogos and derived paraphrasing tools, students can effectively rewrite texts in a non-plagiarized manner. Paraphrasing is a crucial skill, and LLMs and OpenLogos tools can provide multiple paraphrasing suggestions to assist students. While some suggestions may be reliable, others may be inaccurate. Students need to verify the accuracy of the suggestions and select the best option. Through examples provided by LLMs, OpenLogos, and other paraphrasing tools, students can learn and refine their paraphrasing skills.

## The role of LLMs/GenAI and OpenLogos in AI-assisted education

As discussed in Section 2 and Section 3, LLMs and GenAI, as well as OpenLogos can contribute to making education better in a plethora of ways. Teachers can use GenAI to create or adapt educational resources and materials: curricula, lesson materials, code, project ideas and assignments, questions and surveys. With the assistance of GenAI, teachers can easily adapt any instructional material to the age, language proficiency, learning ability and limitations, as well as cultural background. In this regard, LLMs/GenAI can be especially beneficial for adapting materials for students with learning disabilities or neurodevelopmental disorders, ^21– 23^.

The biggest potential of GenAI is that students can themselves use LLMs to adapt the material to their preference and focus more efficiently on problematic parts or areas of interest. For example, students can prompt LLMs to explain difficult concepts in an approachable way, and generate multimodal examples of their real-world application. At a higher level, LLMs can assist students in analyzing, and summarizing research materials for academic projects. Thus, LLMs opens the doors to accessible personalized education. Simulating one-to-one tutoring with a human teacher, LLMs can take students through their educational journey one step at a time, encouraging and motivating them along the way. Ultimately, LLMs can act as career advisers, and help students explore career paths and job opportunities. LLMs can play a significant role in tailoring educational material to different segments of the job market, making it more relevant to students’ career paths. They have the capability to automate the exam process by generating exam content, administering exams to students, and evaluating their performance. This approach ensures thorough and objective assessment while also being time-efficient through parallelization. For students, LLMs can offer practice exams to enhance preparation and identify knowledge gaps. Additionally, they can aid in developing critical thinking skills by prompting students to explain their reasoning. Moreover, LLMs can contribute to improving students’ essay writing and communication skills by providing constructive feedback and suggesting areas for improvement.

LLMs/GenAI and OpenLogos can break language barriers in education by providing real-time translation services for teaching materials and research papers. They are very useful tools especially for those who are conducting research in foreign languages. Numerous universities and individuals have been using OpenLogos for the development of new natural language processing resources and applications, namely a new machine translation system. The capabilities of the OpenLogos resources have been modified and enhanced to create new linguistic resources and new paraphrasing based applications, and are also being used by a multilingual corpora management tool for pattern searching ^33^. Finally, LLMs/GenAI and OpenLogos can assist in the process of second language teaching and language acquisition ^6^. LLMs can provide materials depending on the learner’s language proficiency ^6^. Some learning apps, like Duolingo ^21^ or Grammarly ^22^, have already integrated GPT-4 or other AI technologies.

## Limitations and ethical considerations

As we have already mentioned, LLMs/GenAI have several limitations. They can generate incorrect or misleading information because they tend to hallucinate, and they often lack contextual understanding or real-world knowledge. LLMs/GenAI cannot be a reliable source. Moreover, the performance and the output are dependent on their training data, so there is a variance in the performance between low or mid-resource (like Macedonian) and high-resource languages (English, German, Portuguese). Further studies and surveys should be conducted on the integration of LLMs/GenAI in education. We suggest that the following ethical considerations be taken into consideration:

1. If students take the information (output) for granted, without proper guidance and without checking the information with reliable sources (teacher, books, articles, etc.), adverse knowledge and language transfer may occur. This may induce some other consequences as well.
2. Relying too much on LLMs/GenAI may lead to non-rational behaviors. Students may become dependent on AI technology, which can induce laziness, social isolation, anxiety, depression, a decrease in their IQ and literacy, etc.
3. LLMs/GenAI raise issues around intellectual property rights and facilitate plagiarism among students.
4. LLMs/GenAI do not offer confidentiality and security of data, which may lead to an invasion of privacy.
5. Because of the training data, LLMs/GenAI may generate biased outputs, which can reinforce stereotypes or propagate misinformation or deviations from the truth.
6. LLMs/GenAI might not be equally accessible to all students.

## Conclusions and future work

In this article we explored the influence of NLG, i.e. LLMs/GenAI on the educational sector and the associated research difficulties. We highlighted the impact assessment and the challenges we are facing in the educational settings by illustrating the case study where these systems would be highly beneficial. We also proposed a model of AI integration in education, which presents a structured educational scenario, with three participants: teacher, LLM (AI), and student(s). This type of model can be implemented by any high school, college or university teacher and it can be adjusted to the subject matter. Besides this, we provided several examples about the possible use of the new technologies in education. In the end, we addressed some of the limitations and ethical considerations. Our assessment is only superficial and has different purposes and goals but the same objective of guaranteeing control over information and knowledge. We firmly believe that various methodologies being developed throughout the years driven by a strong linguistic contribution and theoretical background (or combination of backgrounds) can be further explored to increase efficiency, transparency, and public confidence in NLG technology. The integration of LLMs in education and research opens up challenging ethical issues and it raises many questions and concerns. In the realm of LLMs/GenAI, it is crucial to acknowledge the adage that "everything is permissible, but not everything is beneficial". While users have the freedom to explore various topics and ask any questions, not all interactions may be conducive to positive outcomes. It is essential to consider whether our engagements with LLMs contribute positively to our knowledge, understanding, and overall well-being. Effective use of large language models (LLMs/GenAI) requires a high level of emotional, intellectual, and spiritual maturity. Without these qualities, vigilant oversight by teachers, educators, therapists, or parents becomes necessary and even mandatory for proper usage of LLMs. It is important to recognize that while GenAI cannot replace the role of a teacher, when used appropriately, it can enhance learning outcomes and certainly the generation of quality texts. This article can serve as a supplementary resource for studies evaluating the efficacy of language models within educational settings.

## Data Availability

No data are associated with this article.

## Footnotes

^1^ https://openlogos.github.io/

^2^Noam Chomsky proposed the concept of a "universal grammar" and argued that humans possess an innate linguistic capacity, often referred to as the "faculty of language". This concept has been influential in the field of linguistics and cognitive science, shaping our understanding of how language is acquired and processed by the human brain.

^3^Privacy concerns in LLMs encompass various issues, including data security, data collection, data usage, consent and transparency, data retention and deletion, and legal compliance. Data security involves safeguarding learner data from unauthorized access or breaches, while data collection pertains to the types of data gathered by the LLM platform. Data usage raises concerns about how collected data will be used, such as for advertising or user profiling. Consent and transparency focus on informing users about data practices and obtaining their consent. Data retention and deletion involve managing the duration of data storage and user control over their data. Legal compliance ensures adherence to data protection laws and regulations. Addressing these concerns requires robust data protection measures, transparent practices, user consent mechanisms, data control for users, and compliance with relevant privacy laws.

^4^Misinformation refers to false or inaccurate information that is disseminated intentionally or unintentionally, leading to misconceptions, misunderstandings, or harmful outcomes. In the context of LLMs, misinformation may occur when the model generates text that contains factual errors, misleading statements, or unsupported claims. Hallucinations occur when the language model generates text that is nonsensical, illogical, or disconnected from reality. These hallucinations may involve the creation of fictional events, characters, or scenarios that have no basis in truth or coherence. In some cases, hallucinations may result from the model’s tendency to generate plausible-sounding but ultimately meaningless text, especially when prompted with ambiguous or open-ended input.

^5^ www.sciencenews.org/article/chatgpt-ai-artificial-intelligence-education-cheating-accuracy

^6^ www.theguardian.com/us-news/2023/jan/06/new-york-city-schools-ban-ai-chatbot-chatgpt

^7^ www.theguardian.com/technology/2023/may/18/ai-cheating-teaching-chatgpt-students-college-university

^8^ www.techbusinessnews.com.au/blog/chatgpt-may-lead-to-the-downfall-of-eduction-and-critical-thinking/

^9^ www.compilatio.net/en/blog/cheating-chatgpt

^10^ www.wired.com/story/chatgpt-college-university-plagiarism/

^11^ www.washingtonpost.com/education/2023/06/01/students-chatgpt-ai-tools/

^12^ www.euronews.com/next/2023/09/07/first-guide-on-ai-in-education-recommends-13-years-as-minimum-age

^13^The design of Figure 1 was made on Canva, an online graphic design tool.

^14^ https://platform.openai.com/docs/guides/prompt-engineering

^15^ https://guides.libraries.psu.edu/c.php?g=1338692&p=9866353

^16^ www.researchinformation.info/news/cambridge-launches-ai-research-ethics-policy

^17^ www.cambridge.org/core/services/authors/publishing-ethics/research-publishing-ethics-guidelines-for-journals/aut1horship-and-contributorship#ai-contributions-to-research-content

^18^Logos Corporation was a prominent machine translation company that had one of the best models at the time, particularly for its focus on semantic analysis. Logos Corporation’s products were used by various organizations and institutions for translation purposes. The company’s efforts helped advance the field of computational linguistics and contributed to the development of more sophisticated language technologies.

^19^There are 12 parts of speech in SAL, divided into open and closed word classes. Open word classes are: nouns, adjectives, adverbs and verbs. Closed word classes are: articles, prepositions, auxiliary verbs, pronouns, interrogatives, negative particles, conjunctions and punctuation. SAL Taxonomy Tables provide word-specific numeric codes that denote Taxonomy position (superset, set and subset) by their range: Numbers 1–16 denote the superset, 17–99 denote the set, and 100–998 denote the subset.

^20^ Figure 2 shows an example table for the receptacles subset with code 659, that belongs to the functionals set with code 34, which in turn belongs to the CONCRETE Count Noun superset with code 3.

^21^ https://openai.com/customer-stories/duolingo

^22^ www.grammarly.com/ai
