# Supplementary material for: Large Language Models and OpenLogos: An Educational Case Scenario
Source: Open Res Eur. 2024 Jun 5;4:110. [Version 1] doi: 10.12688/openreseurope.17605.1 (PMC11292179; doi:10.12688/openreseurope.17605.1)
Supplement: Peer review history

# Reviewer report: Reviewer response for version 1

**DOI:** 10.21956/openreseurope.19024.r41908

**Published:** 2024-08-05

**Authors:** Nélia Alexandre

**Affiliations:** 1 Universidade de Lisboa, Lisbon, Portugal

**General comment:**

 The article examines the potential benefits and challenges of utilizing Large Language Models (LLMs)/GenAI in an educational setting, emphasising their suitability for classroom use. It addresses pertinent and emerging concerns surrounding the deployment of these novel AI tools and resources, including ethical considerations. By proposing a structured interaction scenario for implementing a Model of LLMs in education, the authors offer valuable guidance on the integration of LLMs in (language) education.

**A few remarks:**

 Footnote 2 (p. 3) provides an explanation of the concept of 'universal grammar' as proposed by Noam Chomsky. It would be appropriate to include at least one reference from N. Chomsky in which he proposes this concept, for example, in 1986, *Knowledge of Language*. [Ref 1]

 It appears that the adverse effects of LLMs in education (see Table 1, p. 4) are more precise than the beneficial effects, which are expressed in a more general manner. A language instructor might inquire as to which aspects of language learning/teaching these LLMs will enhance most effectively, or which innovative teaching methods they could adopt. To be more useful for teaching practitioners, the authors could provide some examples of these effects. This would help to demonstrate how some of the AI tools and resources can be used effectively in the classroom.

 In order to provide a more comprehensive account of the limitations of LLMs and other generative models in the context of language acquisition and teaching, it is necessary to include the term 'teaching' in the sentence “LLMs and other generative models are not entirely adequate for language acquisition/teaching as they…” (p. 5). This is because the models in question are not only unsuitable for acquisition, but also for teaching, and thus this aspect should be explicitly acknowledged.

 The authors posit that the implementation of these technologies could facilitate the creation of more inclusive, ethical, and impactful educational experiences. However, they do not specify how this could be achieved. It would be beneficial for the authors to provide further guidance on this topic, particularly to language instructors who would benefit from more input on this subject.

 Figure 1 (p. 6) provides a comprehensive illustration of the potential applications of LLMs in education, extending beyond the domain of language learning. Nevertheless, it is of limited utility in the context of foreign language learning and teaching. It would be beneficial for the authors to consider providing (in a follow-up paper) an adapted version of Figure 1 that is more suitable for this particular learning/teaching context.

 In the context of foreign language education, the term "real-world language data" (p. 8) is typically used to refer to what is commonly known as "authentic language materials."

 The authors posit that the performance and output of LLMs/GenAI are contingent upon the quality of their training data, with superior results observed in high-resource languages, including English, German, and Portuguese (p.10). It would be interesting to ascertain how these LLMs cope with linguistic variation, particularly in the case of different national varieties of a given language. This is an important issue, given that the number of speakers of a language is often reflected in the amount of data available for that language, with some varieties having a greater number of speakers than others. What will be the impact of this on the maintenance of a less spoken variety?

Is the case presented with sufficient detail to be useful for teaching or other practitioners?

Yes

Is the work clearly and accurately presented and does it cite the current literature?

Yes

If applicable, is the statistical analysis and its interpretation appropriate?

Not applicable

Are all the source data underlying the results available to ensure full reproducibility?

Not applicable

Are the conclusions drawn adequately supported by the results?

Yes

Is the background of the case’s history and progression described in sufficient detail?

Yes

Reviewer Expertise:

Language contact, Second Language Acquisition, Foreign Language Teaching

I confirm that I have read this submission and believe that I have an appropriate level of expertise to confirm that it is of an acceptable scientific standard.

## References

[1] : Knowledge of language. *https://sites.pitt.edu/~perfetti/PDF/Chomsky.pdf* .1986;

# Reviewer report: Reviewer response for version 1

**DOI:** 10.21956/openreseurope.19024.r41906

**Published:** 2024-07-31

**Authors:** Ana Cristina Sousa Martins

**Affiliations:** 1 Centro de Linguística da Universidade Nova de Lisboa, Universidade Nova de Lisboa CLUNL (Ringgold ID: 450876), Lisbon, Lisbon, Portugal

This article aims to provide an overview of the state of the art of NLG applied to AI-assisted education, with a particular focus on paraphrasing, Machine Translation, and second language learning. To this end, the advantages and disadvantages of using LLMs in educational contexts are initially surveyed. The authors contrast LLMs with resources inspired by OpenLogos. Unlike LLMs, which are based on Deep Learning performed through training on large datasets, OpenLogos is a transparent, human-centered generative system involving manual linguistic analysis. Being manually developed by linguists, the coding of OpenLogos can offer access to authentic linguistic data. Additionally, resources inspired by OpenLogos undergo a constant process of testing and experimentation aimed at continuously improving the generated output. Finally, a grounded learning model is suggested that combines LLMs and OpenLogos for verifying the accuracy of automatically generated texts.

**Aspects to Improve:**

1. The article aims at several objectives. It is important to distinguish between primary and secondary objectives.
2. There are several instances where the authors use evaluative expressions (e.g., “a responsible use of AI,” “proper pedagogical guidance,” “use the LLMs and other generative models properly”). It should be briefly explained what is meant by these formulations, as they contain value judgments.
3. The positive and negative effects of using LLMs in an educational context, presented in Table 1, are not of the same order of criteria. For example, «Cheating and Plagiarism» relate to how students use the outputs generated by LLMs, whereas «Bias» corresponds to one of the failures of the outputs themselves. A conceptually differentiated treatment of the advantages and shortcomings of LLMs could be provided.
4. There are concepts that deserve better explanation, such as “paraphrasaries” (p.8).
5. A problem of textual macro-structuring was detected. On page 9, it states: "As discussed in Section 2 and Section 3, LLMs and GenAI, as well as OpenLogos ...". However, OpenLogos is not discussed in these sections.
6. In the conclusion, the authors state that they sought to highlight the impact of the challenges posed by LLMs by presenting a case study. However, the approach to the problem of applying LLMs to education does not constitute a case study as it is not based on any real situation, nor does it involve data collection or analysis. It is possible that what was meant here was a "case scenario". If not, it is important to clarify in what specific sense the term "case study" is being used.

Is the case presented with sufficient detail to be useful for teaching or other practitioners?

Yes

Is the work clearly and accurately presented and does it cite the current literature?

Partly

If applicable, is the statistical analysis and its interpretation appropriate?

Not applicable

Are all the source data underlying the results available to ensure full reproducibility?

Not applicable

Are the conclusions drawn adequately supported by the results?

Yes

Is the background of the case’s history and progression described in sufficient detail?

Yes

Reviewer Expertise:

Text Linguistics and Second/Foreign Language Acquisition

I confirm that I have read this submission and believe that I have an appropriate level of expertise to confirm that it is of an acceptable scientific standard.

# Reviewer report: Reviewer response for version 1

**DOI:** 10.21956/openreseurope.19024.r41907

**Published:** 2024-07-26

**Authors:** Gražina Korvel

**Affiliations:** 1 Vilnius University, Vilnius, Lithuania

The paper examines the role and impact of LLMs and OpenLogos in the field of education. It addresses key issues such as the potential for bias, the need for transparency, and the ethical considerations associated with the use of AI in the classroom. By proposing the integration of OpenLogos with LLMs, the authors present a novel solution to increase the transparency and efficacy of language generation tools.

**A few observations:**

 „faculty of language"2 2 – the reference „2“ is cited twice.

 In "to cheat on school assignments5,6,7,8,9,10,11." a large block of references is grouped together in one place. It would be more effective to list the specific aspect of the claim that each citation supports.

 The use of single quotation marks in the phrase "computer-‘understand’" is unconventional.

Is the case presented with sufficient detail to be useful for teaching or other practitioners?

Yes

Is the work clearly and accurately presented and does it cite the current literature?

Yes

If applicable, is the statistical analysis and its interpretation appropriate?

Not applicable

Are all the source data underlying the results available to ensure full reproducibility?

Not applicable

Are the conclusions drawn adequately supported by the results?

Yes

Is the background of the case’s history and progression described in sufficient detail?

Yes

Reviewer Expertise:

NLP

I confirm that I have read this submission and believe that I have an appropriate level of expertise to confirm that it is of an acceptable scientific standard.
